# Functional ^31^P Magnetic Resonance Spectroscopy at 9.4 T

**DOI:** 10.1002/nbm.70043

**Published:** 2025-04-15

**Authors:** Rolf Pohmann, Julius Schwarz, Klaus Scheffler

**Affiliations:** ^1^ Max‐Planck‐Institute for Biological Cybernetics, Magnetic Resonance Center Tübingen Germany; ^2^ Department of Biomedical Magnetic Resonance University of Tübingen Tübingen Germany

**Keywords:** fMRS, longitudinal relaxation time, phosphorus spectroscopy, ultrahigh field

## Abstract

Functional ^31^P MRS might give valuable information on the energy metabolism of the brain during stimulation. However, the tiny expected spectral changes are difficult to detect due to low SNR and large voxel sizes. Higher field strengths and sensitive multielement arrays could help to investigate the energy metabolism during brain activation. Here, we acquired functional ^31^P MRS data during visual stimulation at 9.4 T using a 27‐element receive array and an optimized 3D‐CSI protocol. First, *T*_1_ values at that field strength were measured, confirming a decrease in *T*_1_ times for increasing fields. Then, a 4.5 min visual stimulus with varying color scheme and frequency was presented to healthy subjects during CSI acquisition for 45 min. Stimulus and rest signals were formed by averaging over five epochs, resulting in spectra with high spectral quality and SNR. Stimulus and rest spectra were almost identical, except for a very small but significant difference in the chemical shift of inorganic phosphate, which might indicate a slight increase in pH during stimulation. It was then investigated whether postprocessing algorithms might be able to detect more subtle changes. Spectral denoising using a principal component approach resulted in improved SNR with high spectral integrity but suppressed the chemical shift difference. Spatial deconvolution improved the spatial resolution but did not yield changes in the outcome. Those results indicate that only very subtle changes in the ^31^P spectrum due to visual stimulation can be expected, which are difficult to detect even when taking advantage of the high SNR and spectral dispersion at 9.4 T.

## Graphical abstract

Functional ^31^P MRS in healthy subjects at 9.4 T was applied to investigate changes in the energy metabolism of the brain during visual stimulation. Stimulation and rest spectra from the visual cortex only differed in a small but highly significant shift in the chemical shift of anorganic phosphate.

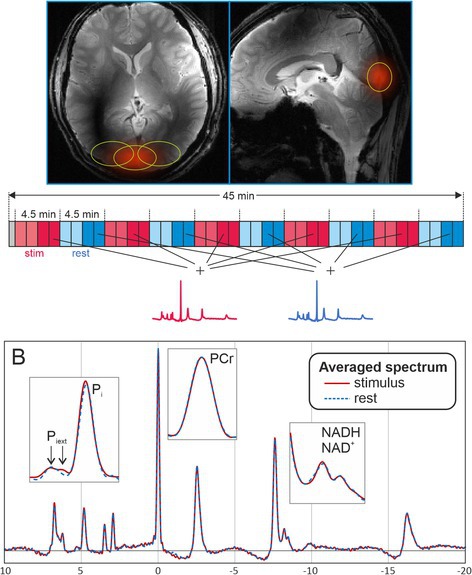

**Abbreviations**
AMARES: advanced method for accurate, robust, and efficient spectral fitting
ATP: adenosine triphosphate
CSI: chemical shift imaging
FOV: field of view
GPC: glycerophosphocholine
GPE: glycerophosphoethanolamine
NAD: nicotinamide adenine dinucleotide
NR: noise reduction strength
OXSA: Oxford Spectroscopy Analysis
PC: phosphocholine
PCr: phosphocreatine
PE: phosphoethanolamine
P_i_
: inorganic phosphate
P_iext_
: extracellular inorganic phosphate
SNR: signal‐to‐noise ratio
SRF: spatial response function
SVD: singular value decomposition
T_I_
: inversion delay
TR‐FOCI: time‐resampled frequency‐offset corrected inversion
UDPG: uridine diphosphate glucose
WSVD: whitened singular value decomposition

## 1 Introduction

Phosphorus MRS allows us to observe the concentrations of some of the main metabolites involved in the supply and consumption of energy in the cells and thus could be able to help in investigating the energy metabolism of healthy and diseased organs. In the skeletal muscle, the effects of increased energy consumption due to exercise have been shown to be clearly visible in the phosphorus spectra as a drastic reduction in the concentration of phosphocreatine (PCr) as an energy buffer, while the concentration of inorganic phosphate (P_i_) grows and its chemical shift changes due to a decrease in pH during the exercise [1, 2]. It is an obvious question whether we might be able to observe similar effects when increasing the energy consumption in the brain by neuronal activation. Accordingly, several studies have tried to detect changes in phosphorus spectra of the healthy [3, 4, 5, 6, 7, 8] and diseased [9, 10, 11] human brain during—mainly visual—stimulation, however, with inconsistent and strongly varying results. More recent studies at 7 T [12, 13, 14] find no [13] or only slight modifications in the P_i_ [12] or the nicotinamide adenine dinucleotide (NAD) [14] peaks.

Higher field strengths can be especially helpful to improve the significance of ^31^P spectroscopic measurements, as the increase in sensitivity [15, 16, 17] and the improved spectral dispersion at higher fields are accompanied by a decrease of *T*_1_, which further helps to increase SNR [18]. The aim of this study, thus, is to use the advantages of phosphorus spectroscopy at 9.4 T in combination with a highly sensitive receive coil array, an optimized protocol, and modern postprocessing techniques to elucidate the effect of visual stimulation on the energy metabolism as observed by ^31^P spectroscopy.

This study consists of three steps. First, the longitudinal relaxation times of the ^31^P peaks in the visual cortex at 9.4 T are determined, as this information is required to obtain SNR‐optimized measurements. Second, the functional study is performed, and the data is analyzed for changes between stimulated and rest conditions. And third, the effect of denoising and spatial deconvolution on the outcome is examined.

## 2 Methods

All spectra were acquired on a 9.4 T MR scanner (Siemens Healthcare, Erlangen, Germany) equipped with a whole body gradient (SC72, Siemens). The home‐built double‐tuned head coil consisted of four large loops for ^31^P‐transmit in circularly polarized mode, combined with 27 overlapping receive loops with a diameter of 7 cm in four rows on a helmet‐shaped holder to minimize the distance between the coil and the head. In addition, four transmit/receive proton dipole elements were used for localization and *B*_0_‐shimming [19]. A total of 22 healthy volunteers were scanned after informed consent and with approval by the local ethics board.

The subject was placed in the magnet, and proton images were acquired for localization. A region encompassing the visual cortex was used for local *B*_0_‐shimming using a vendor‐supplied, field map–based technique. The transmitter reference power was set to a value determined beforehand in several subject measurements. Then, one of two sets of measurements was performed:

### 2.1 T_1_‐Quantification

To optimize sequence parameters, knowledge of relaxation times is necessary. *T*_1_ was measured with an inversion recovery sequence on six subjects using a 15 ms adiabatic TR‐FOCI inversion pulse [20] followed by a nonselective single‐pulse spectroscopic readout. Eight slice‐selective saturation pulses immediately before the excitation were applied to suppress signal from outside the visual cortex. Seven or eight inversion delays (*T*_*I*_) between 61 ms and 14 s were acquired with a repetition time (*T*_*R*_) of 15 s and 20 averages for each *T*_*I*_. The data for the longest *T*_*I*_ of 14 s was scanned with 40 averages to obtain a spectrum with very high SNR as a reference for quantification. A nominal bandwidth of 5000 Hz was digitized with 256 samples within 51 ms. With a nonselective excitation pulse of 0.3 ms, an acquisition delay of 0.25 ms between the center of the pulse and the start of the acquisition was reached. To improve localization, only the signals acquired with the two receive elements positioned below the visual cortex were phased and combined for reconstruction and analysis of the signals.

The first six points of each FID were removed to improve the spectral baseline by suppressing broad baseline peaks from short‐lived signals. The data from the two channels were manually phased and added. The AMARES [21] algorithm of the jMRUI [22] software package was used to quantify all spectra by fitting Lorenzian line shapes to nine spectral lines assigned to phosphoethanolamine (PE), phosphocholine (PC), P_i_, glycerophosphoethanolamine (GPE), glycerophosphocholine (GPC), PCr, γ‐adenosine triphosphate (γ‐ATP), α‐adenosine triphosphate (α‐ATP), and a single peak for NAD. The peak of β‐ATP is not quantified as its chemical shift may be too large to be fully excited due to the limited bandwidth of the rf pulses. The data with the longest inversion time and a higher number of averages were fitted first to find accurate values for linewidths and frequencies that were then used as prior knowledge in the AMARES analysis of the other spectra. The *T*_1_ values of the inversion recovery series were determined by fitting a monoexponential inversion–recovery curve $S=M_{0}\left(1-2\cdot c\cdot e^{-T_{I}/T_{1}}\right)$ to the amplitudes *S* determined by AMARES, where *M*_0_ (equilibrium magnetization), *c* (inversion efficiency), and *T*_1_ were the fitted parameters. In addition, the *T*_2_* values of the individual metabolites were determined from the linewidths found by the jMRUI analysis of the longest inversion delay.

### 2.2 ^31^P fMRS

**FIGURE 1:**
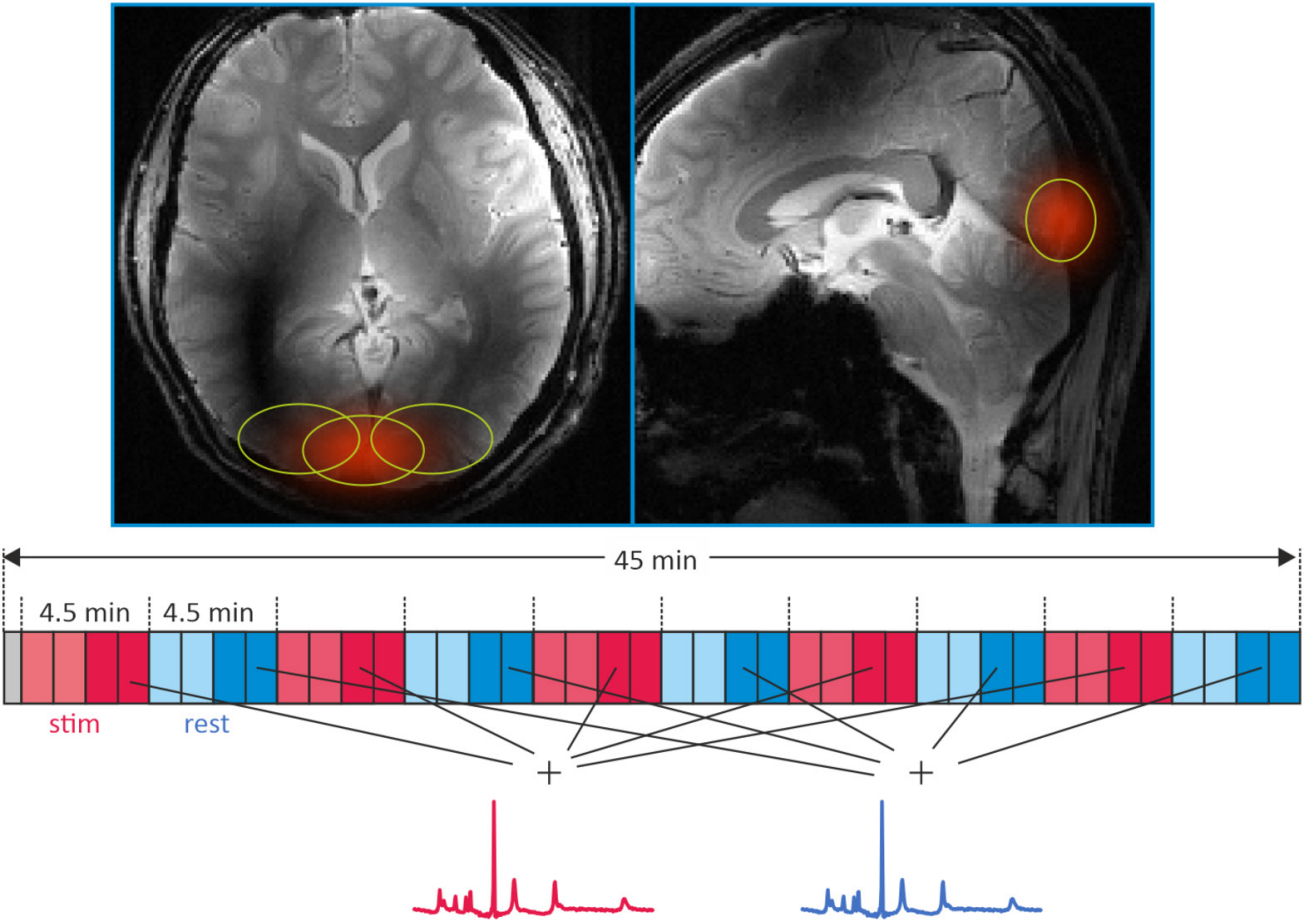
(Top) fMRS voxel positions for one subject. Three nonisotropic voxels inside the visual cortex are selected (yellow ellipses delineating 64% lines). For the center voxels, the spatial response function is plotted in red. (Bottom) Stimulation paradigm and CSI timing. Five pairs of rest/visual stimulation periods of 4.5 min each are filled with four 3D‐CSI acquisitions each. For analysis, the last two scans from each period were added to form one stimulus and one rest spectrum.

In the functional experiments, a 3D‐CSI sequence was used to avoid the chemical shift displacement effects unavoidable in most volume selection techniques at this field strength [23]. For each of 16 subjects (age 29 ± 10 years, six female), 40 CSI scans were acquired, each consisting of 7 × 11 × 11 phase encoding steps with an optimized k‐space weighting over a FOV of (180 mm)^3^, leading to a nonisotropic nominal voxel size of 25.7 × 16.4 × 16.4 mm^3^. The real voxel size, as determined from the width of the spatial response function (SRF) was 41 × 24 × 28 mm^3^, with the nonisotropic voxels oriented to align with the shape of the cortex in the expected activated volume (see Figure 1). The voxel volume, defined as the region where the SRF is above 64% of its maximum, was 14.9 cm^3^. With a repetition time of 62 ms and up to eight averages in the k‐space center, each CSI scan took 68 s, leading to a total experiment duration of around 45 min. The flip angle was set to 14°, in the range of Ernst angles for the main metabolites of interest based on the *T*_1_ times measured above.

The stimulation paradigm consisted of five repetitions of a 4.5 min flickering checkerboard, followed by 4.5 min of rest, corresponding to four repetitions of the CSI sequence per stimulation/rest period (Figure 1). To avoid adaptation effects during this relatively long stimulus [24], the color scheme of the checkerboard was varied seven times per stimulation period [25], while the frequency of flickering [26] was varied 16 times in eight steps between 2.2 and 8 Hz in random order. Subject attentiveness was enhanced and verified by showing a central fixation cross and asking the subject to press a button whenever this cross changed color. The stimulus was created with PsychoPy [27], projected onto a screen positioned at the back of the magnet bore and observed by the subject via a mirror inside the coil, right above the eyes.

Postprocessing started with a WSVD (whitened singular value decomposition)‐based coil combination [28], taking only the 10 receive coils around the visual cortex into account, and a weighting correction to compensate for the integer‐only k‐space weighting. Then, a high‐SNR dataset was constructed by averaging all 40 repetitions. In addition, a pair of stimulus/rest spectra was generated by adding the last two scans of all stimulus and rest periods, respectively (see Figure 1). The first two scans per period were discarded to allow some time for spectral changes to develop. The resulting datasets, as well as the high‐SNR dataset, were zero‐filled to 48^3^ voxels and Fourier‐transformed. Anatomical images were used to select three voxels in the visual cortex, positioned in the left and right hemispheres and at the center (Figure 1), from which the data was taken for analysis. In addition, signals from three control voxels in the cerebellum, the temporal, and the parietal lobes were analyzed. For the spectra displayed in the figures, the missing spectral points at the beginning of each FID due to the time needed for phase encoding were extrapolated using Burg's method [29] and zero filled. This processing step was not applied to those spectra that were quantified with AMARES, as we found that the fitting quality was degraded after extrapolation. No exponential filtering or other signal manipulations were applied. All spectra are displayed with the PCr peak set to a chemical shift of zero.

For quantification, a MATLAB implementation of the AMARES [21] algorithm included in the OXSA (Oxford Spectroscopy Analysis) toolbox [30] was used in a multistep approach. First, the high‐SNR spectrum was fitted to obtain highly accurate values for all spectral parameters of all peaks. Then, the stimulation and rest spectra were quantified, where the results of the high‐SNR fit were used as initial values. In addition, linewidths (except for PCr and P_i_) and chemical shifts (except for P_i_ and extracellular/mitochondrial inorganic phosphate (P_iext_)) were fixed to the values found in the high‐SNR spectra, while all amplitudes (area under the peaks) were determined with AMARES. Thirteen metabolites were quantified (PCr, β‐ATP, α‐ATP (doublet), γ‐ATP (doublet), P_i_, GPC, GPE, PC, PE, NADH, NAD^+^ (reduced and oxidized form of NAD), uridine diphosphate glucose (UDPG), and P_iext_). The parameters for fitting the two NAD components closely followed previous studies [31, 32]. All amplitudes were normalized by the PCr amplitude (mean value of fitting result of rest and stimulus spectra). The determined spectral parameters were then tested for differences between stimulus and rest using a paired *t*‐test. Bonferroni’s correction was applied to account for the large number of compared parameters. For the analysis presented here, the three spectra acquired from each subject were treated as individual data points.

### 2.3 Denoising and Spatial Deconvolution

To investigate whether improving the spectral quality by applying additional postprocessing steps can help to uncover still invisible peak changes, we first investigated the influence of a singular value decomposition (SVD)–based denoising technique [33] on the outcome, closely following the procedure described previously [34]. Unaveraged signals from a 3 × 3 × 3 × 40 voxel patch around each voxel and through all scans were pooled and subjected to a SVD. Then, only 1/NR of the singular values were retained, where the noise reduction strength NR was varied within [2,4,8,16,32,64]. The spectra were then reconstructed from the remaining singular values, averaged across rest and stimulation states, and processed and analyzed as above.

As subtle changes in the metabolite concentrations may disappear due to partial volume effects caused by the large voxel sizes, a retrospective reduction of the voxel volume by spatial deconvolution [34] was applied both alone and in combination with the noise reduction. For this, a Wiener filter [35] in its inbuilt MATLAB R2022a implementation was used, again similar to the procedure described previously [34]. The filter was applied to each point of the magnitude of the temporal signal after spatial Fourier transformation and zero filling, taking the point‐spread function of the k‐space weighted measurement into account. The phase of the original signal was then reapplied to the filtered data. The deconvolution parameters were adjusted to yield a reduction of the voxel volume by approximately one half.

## 3 Results

### 3.1 Relaxation Times

**FIGURE 2:**
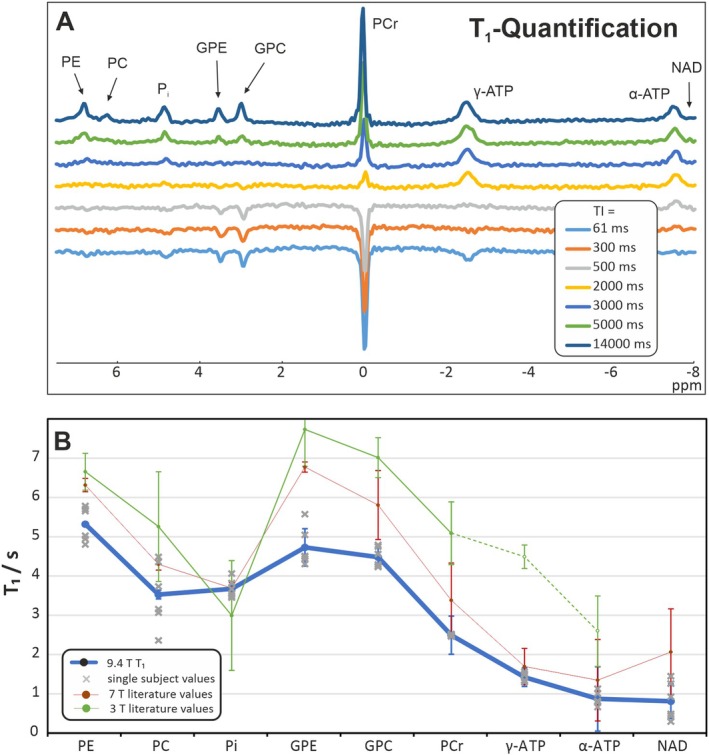
(A) Inversion‐recovery spectra from one subject for all seven inversion times. (B) Measured *T*_1_‐values at 9.4 T in comparison to literature data from 7 T [36] and 3 T (combined from [37, 38, 39], points connected with broken line are from calf muscle). For the 9.4 T data, the single values from all six subjects are shown as well.

Excellent spectral quality was obtained from all subjects, allowing jMRUI fits with small error margins for most resonances (Figure 2A). Monoexponential fits resulted in the *T*_1_‐values listed in Table 1 and shown in Figure 2B in comparison to published values from 7 T [36] and 3 T [37, 38, 39]. As several studies [40, 41] in animals have found unchanged *T*_1_‐times for varying physiological states, we assume these values to be valid for both rest and activation.

**TABLE 1:** Measured values for the relaxation times *T*_1_ and *T*_2_*.

	*T*_1_/ms	*T*_2_*/ms
PE	5333 ± 441	8.78 ± 1.06
PC	3536 ± 821	8.49 ± 2.33
P_i_	3683 ± 234	10.2 ± 0.96
GPE	4740 ± 487	15.7 ± 1.6
GPC	4491 ± 255	13.6 ± 1.9
PCr	2499 ± 28	21.2 ± 3.7
γ‐ATP	1426 ± 111	5.72 ± 0.24
α‐ATP	872 ± 170	6.97 ± 0.43
NAD	774 ± 519	8.23 ± 1.9

### 3.2 ^31^P fMRS

**FIGURE 3:**
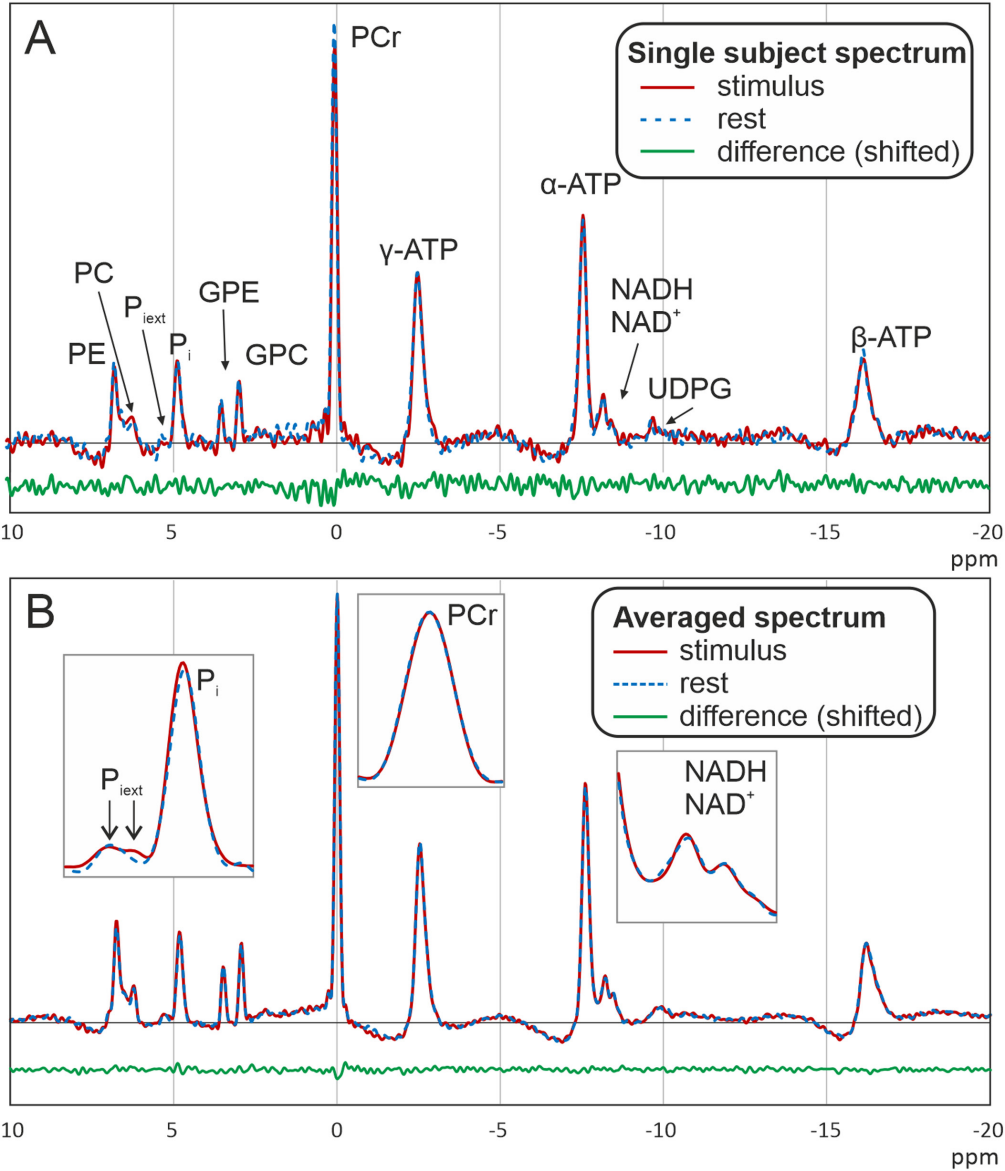
(A) Rest and activation spectrum from one subject with two averages per rest/stimulation period as shown in Figure 1. (B) Rest and activation spectra, averaged over 15 subjects. The inserts show zoomed reproductions of some of the most interesting regions.

Figure 3A shows stimulation and rest spectra from the same voxel of the same subject, demonstrating the excellent spectral quality in all measurements. For one subject, there were strong amplitude variations between the single scans, which might have been caused by motion. This subject was discarded in all further analyses. For the remaining 15 subjects, the AMARES fitting routine yielded high fitting quality with low residuals. For the analysis, the three spectra acquired from each subject were treated as individual data points, yielding a total of 45 pairs of analyzed spectra.

As shown in Figure 3A, spectra acquired during stimulus and rest largely coincide. Accordingly, for most quantified parameters, no significant differences between the two conditions were found. Mean values of all quantified parameters at rest and stimulus, including the *p*‐values for the *t*‐test, are shown in Table 2. After Bonferroni correction, the only significant difference was the chemical shift of P_i_, which shows a tiny (1.2 Hz) but highly significant (*p* = 5.4·10^−6^) increase with a medium statistical effect size (Cohen's *d*) of 0.4. The quantification results of P_iext_ and UDPG show high variability due to low SNR (standard deviation more than half of amplitude value) and were therefore ignored in the quantitative evaluation.

**TABLE 2:** Quantification results for the fitted parameters for rest and stimulation. Values given are the mean over 15 subjects ± standard deviation. Amplitude values are given as fitted amplitude divided by the mean of stimulus and rest values for PCr. The last column lists the *p*‐values for a comparison of rest and stimulus values as found in a paired *t*‐test. After Bonferroni correction (17 comparisons, *p* < 0.0029 for significance), only the chemical shift of P_i_ shows a significant difference between the two conditions.

	Metabolite	Rest	Stimulation	*p*
Linewidth (Hz)	PCr	23.1 ± 2.9	23.4 ± 2.8	0.35
	P_i_	31.1 ± 4.1	31.4 ± 3.9	0.46
Chemical shift (ppm)	P_i_	4.81 ± 0.03	4.82 ± 0.03	**5.4·10** ^**−6**^
	P_iext_	5.31 ± 0.09	5.32 ± 0.08	0.46
Relative amplitude	PCr	1.00 ± 0.03	1.00 ± 0.03	0.48
	α‐ATP	0.94 ± 0.20	0.94 ± 0.20	0.48
	γ‐ATP	1.00 ± 0.25	1.00 ± 0.24	0.73
	P_i_	0.28 ± 0.06	0.29 ± 0.07	0.05
	GPC	0.18 ± 0.04	0.18 ± 0.05	0.15
	GPE	0.14 ± 0.04	0.14 ± 0.04	0.39
	PC	0.18 ± 0.06	0.19 ± 0.06	0.12
	PE	0.38 ± 0.10	0.40 ± 0.12	0.01
	NADH	0.09 ± 0.04	0.10 ± 0.03	0.32
	NAD^+^	0.19 ± 0.05	0.18 ± 0.06	0.52
	UDPG	0.06 ± 0.05	0.06 ± 0.03	0.41
	P_iext_	0.03 ± 0.03	0.04 ± 0.03	0.28
	NAD^+^/NADH	2.8 ± 2.9	2.2 ± 1.4	0.11

Figure 3B shows rest and activation spectra averaged over all 15 subjects after phase adjustment to obtain very high SNR. The insets contain some peaks that are of special interest. For the PCr peak, a change neither in amplitude nor in the linewidth is visible. In P_i_, the frequency shift is barely visible; an apparent difference in amplitude is not significant. For the P_iext_ peak, a shifted component appears to be visible in the stimulus spectrum; however, this is close to the noise level and cannot be quantified with sufficient certainty. The NAD peaks do not appear changed between the two conditions. In the control voxels outside of the visual cortex, no significant differences between stimulation and rest were found.

**FIGURE 4:**
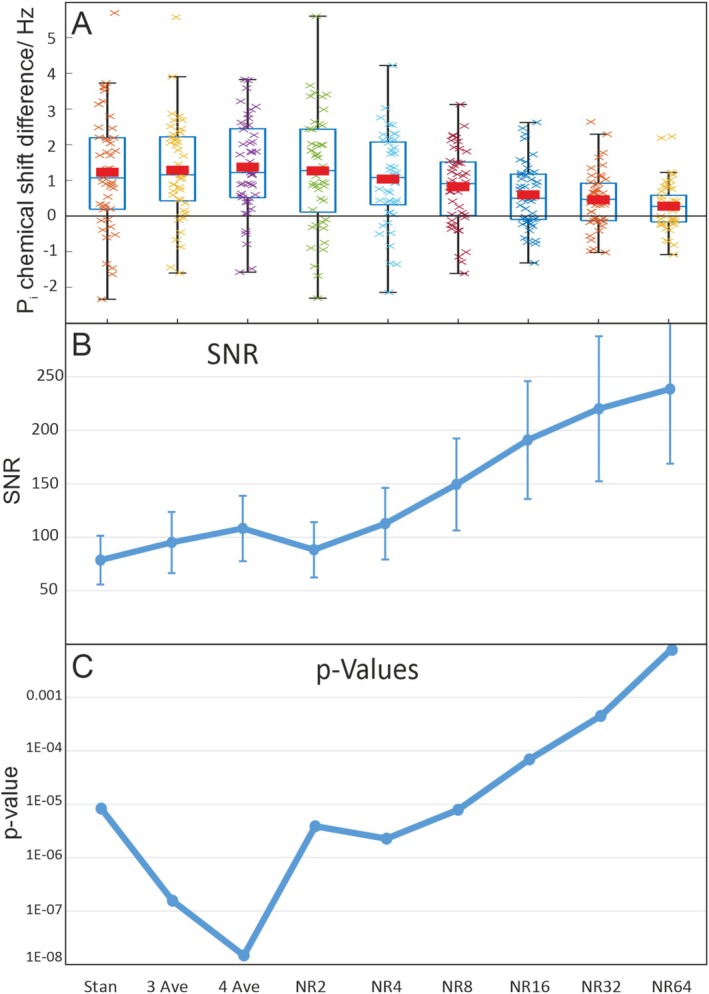
The effect of averaging (two to four averages per epoch) and noise reduction (NR = 2–64) on the fMRS spectra. (A) P_i_ chemical shift difference between stimulation and rest. Shown are box plots with mean values (red bars) and single measurement values. (B) Spectral SNR of single subject/voxel spectra, calculated as maximum of the PCr peak divided by standard deviation of a peak‐free region. (C) *p*‐values for paired *t*‐tests between the chemical shifts of P_i_ at rest and stimulus.

The experimental protocol used allowed us to increase SNR at the cost of the time allowing metabolite changes to occur by averaging three or even all four scans per stimulus and rest period instead of only using the last two measurements per epoch (Figure 4B). This results in significantly higher SNRs and improved fitting results with reduced residuals. However, the quantification results change only slightly. Still, only the P_i_ peak shows a significant difference between rest and stimulation. In addition to its frequency (*p* = 1.6e‐7/1.5e‐8 for three and four averages, respectively), however, there is also a significant difference in the amplitudes of the P_i_ peak (rest: 0.28 vs. stim: 0.30, *p* = 0.0002 for three averages and rest: 0.28 vs. stim: 0.30, *p* = 3.6·10^−5^ for four averages).

Similarly, we can investigate differences between the first and subsequent stimulations by averaging all four scans of each stimulation and rest period separately. However, we do not find differences between spectra acquired during the first and the later stimuli. In addition, no additional significant differences between stimulus and rest can be detected for the first stimulus, again showing the frequency of the P_i_ peak as the only significant difference. After averaging over all 15 subjects, no obvious differences between first rest and first stimulus or between first and second stimuli can be seen.

Finally, separately averaging the four timepoints during each stimulation/rest period allows us to observe changes during the stimulus and rest periods in a temporally resolved manner. However, no temporal evolution is visible for P_i_ frequency and amplitude. P_i_ frequency is again significantly different between stimulus and rest for all timepoints (*p* < 0.002) but does not show systematic variations over the single epochs. Similarly, the P_i_ amplitude appears systematically higher during stimulus for all timepoints; however, this difference is not significant.

### 3.3 Noise Reduction

**FIGURE 5:**
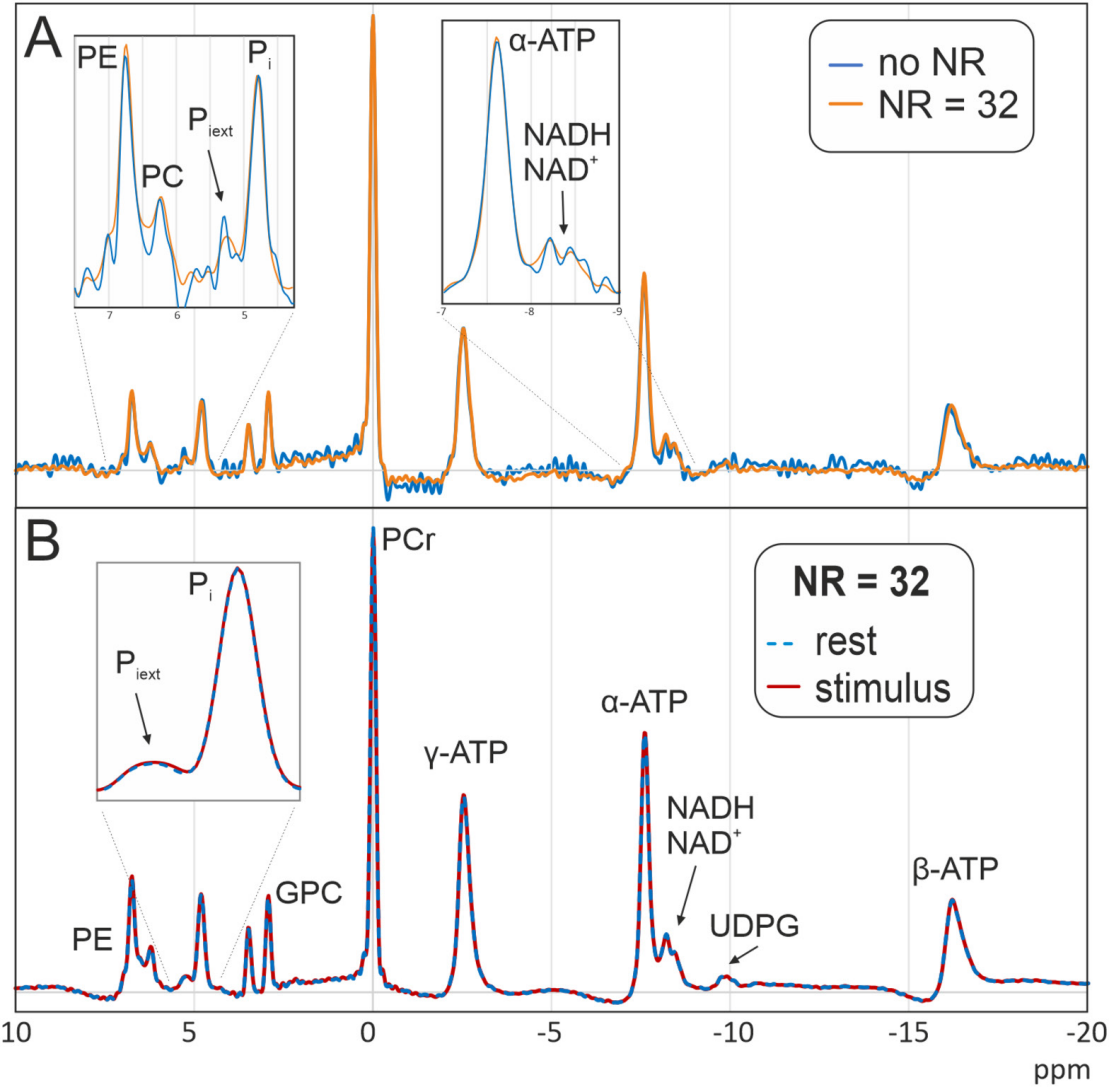
Effects of noise reduction. (A) Comparison of a spectrum from one subject with and without noise reduction with NR = 32. All spectral lines show close agreement with and without noise reduction. (B) After noise reduction with NR = 32, rest and stimulus spectra appear equal.

Stimulation and rest spectra processed with the different noise reduction levels show an SNR increase with increasing NR strength (Figure 4) while the spectral appearance remains very similar for NR up to 64. As the directives from the original publication [33] indicate an NR of 32 as optimum to remove noise and retain information, based on the Marchenko–Pastur distribution, this value is used in most of the further results. To assess changes to the spectra due to the noise reduction, spectra averaged over all 15 subjects and three voxels each, with and without noise reduction with NR = 32, are compared in Figure 5B. The two spectra overlap almost perfectly, indicating high spectral integrity of the noise reduction process. A closer look at single voxel spectra from one subject and voxel (Figure 5A) again shows a very high agreement in the shapes of the most prominent peaks. However, the small resonances, especially GPC, PC, NADH, and NAD^+^, demonstrate some differences in linewidth and amplitude, which may influence the quantification (Figure 5A, insets). Accordingly, differences in the quantified amplitudes of the larger peaks between the spectra with and without noise reduction (NR = 32) remain below 5%, while the weaker signals show changes of up to 10%.

Most prominently, however, is a reduction in the difference of the chemical shift of P_i_ between rest and stimulation with increasing NR strength, caused by shifts of this peak towards the common mean, which causes the significance of the frequency change to disappear with higher NR strengths (Figure 4).

### 3.4 Spatial Deconvolution

**FIGURE 6:**
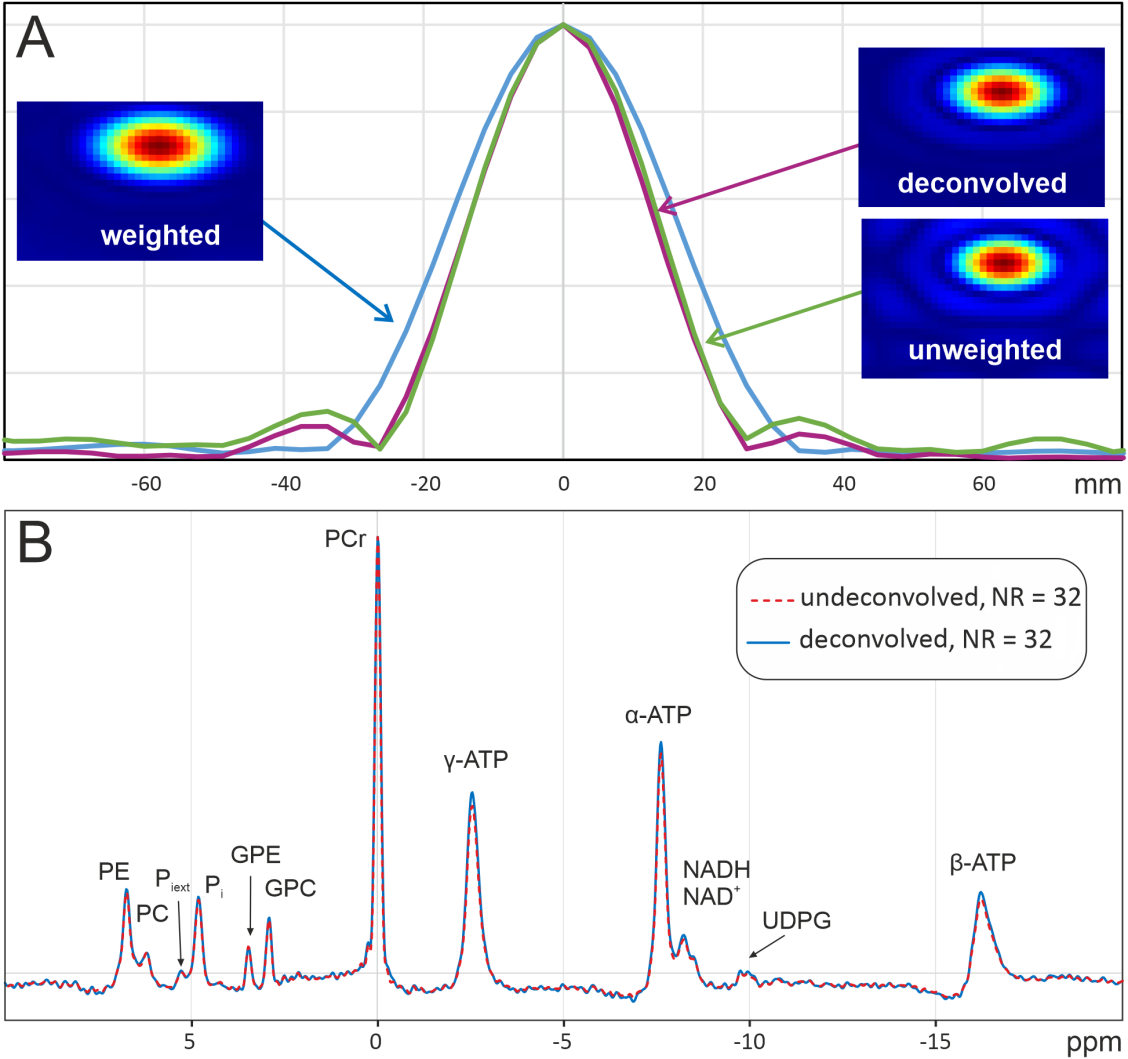
Spatial deconvolution. (A) Signal distribution of a small sample with and without *k*‐space weighting and after spatial deconvolution. (B) Single subject spectra with and without deconvolution.

A possible reason for the lack of a more distinct effect of the stimulation on the ^31^P spectrum is the large voxel size, which may cause significant dilution of the signal as the effectively activated region may only be a small fraction of the entire voxel. While the reduced SNR prevents smaller voxel sizes for unprocessed signals, higher spatial resolutions may become possible by applying noise reduction. Retrospectively reducing the voxel size based on knowledge of the point spread function is possible with deconvolution algorithms. In our analysis, the regularization parameter was chosen such that the total voxel volume was approximately halved. The effect of the filter on the point spread function can be assessed by regarding the signal of a small sample filled with phenylphosphonic acid that was placed above the head of the subject in some measurements. Figure 6A compares the image and the spatial distribution of its signal between the original weighted measurement, the deconvolved version, and the signal of an unweighted measurement. The deconvolved signal has closely the same extent as the unweighted one, though with reduced Gibbs ringing, connected to a voxel volume of 53% of that of the original weighted measurement.

As the deconvolution also reduces SNR, it is most reasonably applied together with noise reduction. An example of a deconvolved spectrum in comparison to an original one with a NR of 32 is displayed in Figure 6B, where a very good agreement between the two signals is visible. Changes in the quantified amplitudes were below 6% for all peaks except for the NAD resonances, where a mean deviation of up to 10% was reached. While the chemical shift difference of P_i_ was still significant for the non‐noise reduced deconvolved spectra, this difference disappeared after noise reduction in the same way as for the undeconvolved signals.

## 4 Discussion

X‐nuclei spectroscopy is one of the applications that may profit most from using higher fields, as it takes advantage of both the improved SNR and the increased spectral dispersion. Previous studies at 9.4 T [23, 42, 43] have already demonstrated the high spectral quality that can be reached at this field strength. In combination with a highly sensitive receive coil array, we were able to obtain spectra with a high SNR from a relatively small voxel volume of less than 15 cm^3^. In addition, the advantages of the high field can be appreciated in the clear separation of the NAD peaks, which allows a relatively good quantification of those peaks, especially after noise reduction. In addition, the P_iext_ peak is clearly visible in many of the subjects, even though its amplitude is not always sufficient for reliable quantification.

Our *T*_1_‐measurements confirmed the observation of a decreasing *T*_1_ with increasing field for this nucleus, indicating a strong influence of chemical shift anisotropy on the relaxation and, by further increasing SNR, forming an additional benefit of higher field strengths.

However, in spite of those advantages, we were not able to detect distinct activation effects on the ^31^P spectrum. This is surprising, as magnetization transfer experiments showed an increasing activity of creatine kinase and ATPase [39, 44], which indicate enhanced energy consumption. Especially, we did not see the effect of BOLD, which should be visible as a change in the linewidth of the PCr line, indicating a slower local *T*_2_* decay during activation. However, only a small change in linewidth is expected. In proton spectroscopy at 7 T, a reduction in linewidth of 0.48 Hz was observed [45], which would translate to 0.26 Hz for ^31^P at 9.4 T due to the different gyromagnetic ratios and field strengths, which might be too small to be detectable. Furthermore, the still relatively large size of the voxels may cause a loss of sensitivity to activation effects, as it may contain much more nonactivated than activated regions. Reducing the voxel size by Wiener deconvolution in combination with noise reduction did not help to uncover the BOLD effect, suggesting that much higher spatial resolutions might be necessary. If, however, the BOLD effect is obscured by large signal contributions from nonactivated tissue, small activation effects on other spectral features may similarly become invisible due to the large voxel size.

The only effect of the activation we were able to quantify is a small upward shift in the frequency of P_i_, which is highly significant but has a very limited size of only around 1 Hz, corresponding to an increase in pH [46, 47] by less than 0.01. Furthermore, this effect is reduced when applying noise reduction, while increasing the SNR of the spectra by enhanced averaging increases the frequency shift. Currently, little is known about the size and direction of a potential pH shift during activation. Previous measurements have found a reduction in pH of about 0.01 at 3 T [48], while more recent measurements at 7 T set an upper limit at 0.03, as no effect could be observed at this measurement precision [49]. Theoretically, processes for changes in both directions are possible [50]. However, in a study [51] at 7 T, which also found a significant increase in pH of about 0.01 during stimulation, this finding is attributed to the increased consumption of H^+^‐ions due to elevated ATP production during stimulation.

Another resonance of interest is that of P_iext_, which, unfortunately, is too weak for reliable quantification. After averaging over all subjects, a second resonance with a lower pH seems to become visible, which, however, is not clearly above the noise level. The visibility of this peak also suffers from the fact that the P_iext_ line is not well visible in all subjects. Only selecting subjects where this peak is well defined yields a much clearer resonance at a pH of about 7.34 during activation in addition to the normal P_iext_ peak at a pH of around 7.55 in rest and activation spectra, which might indicate an effect on one phosphate pool. While this agrees with previous observations at 7 T [12], the limited amplitude of this peak does not allow a definite conclusion. A signal appearing here might even be due to an increase in the signal of 2,3‐DPG due to increased blood volume [52, 53].

For the results presented here, the data from three voxels inside the visual cortex of each subject were treated as separate data points, yielding a total of 45 rest/activation pairs. Alternatively, the three spectra per subject can be averaged before quantification, with a very similar outcome. Comparing spectra from the center, left, and right visual cortex individually over all subjects still results in significant differences for the chemical shift of P_i_ at the 5% level, but not at the lower level required by Bonferroni correction, due to the lower number of data points.

To help in further improving the spectral quality to be able to detect even smaller changes, we investigated the effect of advanced postprocessing routines on the results. SVD‐based denoising resulted in improved SNR with high spectral integrity. However, minor variations in the shape and amplitude of the smaller resonances were visible, which caused the differences between activated and rest spectra to disappear. As no ground truth is known, no definite conclusions can be drawn. Other noise reduction techniques [54, 55, 56] have shown excellent results in ^31^P MRS and could be alternatives to the method used here. After averaging over all subjects, the SNR even without noise reduction is excellent and not perceptibly different from that of the corresponding noise‐reduced spectra. However, the frequency shift as well as the shifted P_iext_ peak in the stimulation spectrum is still visible, while it disappears after noise reduction (compare insets in Figures 3B and 5B). Furthermore, improving the SNR by increasing the number of averages retains or even enhances the observed activation effect, which makes it plausible that this effect is real and not only a chance result of fitting noisy spectra. This indicates that noise reduction should be applied carefully if very small effects are expected.

While we applied relatively long stimulation periods of 4.5 min, longer stimulations, stronger stimuli, or different stimulation paradigms may have a larger effect and generate stronger modulations of the ^31^P spectra. Furthermore, several studies have found elevated reaction rates of both the ATPase and the creatine kinase reactions during stimulation, using a magnetization transfer approach [39, 51]. While this kind of experiment will become more difficult at higher fields due to the increased SAR, the higher SNR and spectral dispersion at 9.4 T might yield more accurate values of those parameters.

The small extent of changes between rest and activity in the ^31^P spectra is an indication of the high level of homeostasis in the brain cells. While all our measurements were performed on healthy and mostly young subjects, disturbed energy homeostasis may elicit stronger changes in the metabolic profile. It would therefore be of interest to try similar measurements in aging subjects or patients suffering from pathologies like Parkinson's [57] or hepatic encephalopathy.

As the large voxel size used in ^31^P spectroscopy may obliterate potential activation effects due to partial volume effects by mixing the signal from small activated regions with that of large, nonactivated areas, higher spatial resolutions may be crucial for functional MRS. Spatial deconvolution can be an important means for retrospectively reducing the voxel size, especially in combination with noise reduction techniques to recover the SNR lost by improving the spatial resolution. Our measurements showed a reduction of the voxel size by a factor of around two with reduced Gibbs ringing compared to unweighted CSI and high spectral integrity. However, even this reduction in resolution did not help to uncover activation effects on the ^31^P spectrum.

## 5 Conclusion

In spite of the high spectral quality at the high field strength, only minor variations of the ^31^P spectrum due to activation could be observed. This indicates that, even for relatively strong visual stimulation, no drastic changes in metabolism or depletion of energy reserves are necessary. To observe the effects of the stimulation on the phosphorus spectrum will probably require significantly improved spatial resolution to reduce partial volume effects. This, however, will be difficult due to insufficient SNR.

## Data Availability

The data that support the findings of this study are available from the corresponding author upon reasonable request.
